# Laparoscopic versus open hepatic resection in patients ≥75 years old: A NSQIP analysis evaluating 2674 patients

**DOI:** 10.1002/jso.27820

**Published:** 2024-08-19

**Authors:** Kevin Verhoeff, Juan Glinka, Douglas Quan, Anton Skaro, Ephraim S. Tang

**Affiliations:** ^1^ Department of Surgery University of Alberta Edmonton Alberta Canada; ^2^ Department of Surgery University of Western Ontario London Ontario Canada

**Keywords:** geriatric liver resection, hepatectomy, laparoscopic liver resection, liver resection

## Abstract

**Background:**

Previous studies report promising outcomes with minimally invasive (MIS) hepatectomy in elderly patients but remain limited by small size. This study aims to comparatively evaluate the demographics and outcomes of geriatric patients undergoing MIS and open hepatectomy.

**Method:**

The 2016–2021 NSQIP database was evaluated comparing patients ≥75 undergoing MIS versus open hepatectomy. Patient selection and outcomes were compared using bivariate analysis with multivariable modeling (MVR) evaluating factors associated with serious complications and mortality. Propensity score matched (PSM) analysis further evaluated serious complications, mortality, length of stay (LOS), Clavien Dindo Classification (CDC), and Comprehensive Complication Index (CCI) for cohorts.

**Results:**

We evaluated 2674 patients with 681 (25.5%) receiving MIS hepatectomy. MIS approaches were used more for partial lobectomy (85.9% vs. 61.7%; *p* < 0.001), and required fewer biliary reconstructions (1.6% vs. 10.6%; *p* < 0.001). Patients were similar with regards to sex, body mass index, and other comorbidities. Unadjusted analysis demonstrated that MIS approaches had fewer serious complications (8.8% vs. 18.7%; *p* < 0.001). However, after controlling for cohort differences the MIS approach was not associated with reduced likelihood of serious complications (odds ratio [OR]: 0.77; *p* = 0.219) or mortality (OR: 1.19; *p* = 0.623). PSM analysis further supported no difference in serious complications (*p* = 0.403) or mortality (*p* = 0.446). However, following PSM a significant reduction in LOS (−1.99 days; *p* < 0.001), CDC (−0.26 points; *p* = 0.016) and CCI (−2.79 points; *p* = 0.022) was demonstrated with MIS approaches.

**Conclusions:**

This is the largest study comparing MIS and open hepatectomy in elderly patients. Results temper previously reported outcomes but support reduced LOS and complications with MIS approaches.

## 1 INTRODUCTION

Hepatectomy (i.e., hepatic resection) for both primary and metastatic liver lesions continues to expand.^1^, ^2^ Similarly, the proportion of elderly patients requiring hepatic resection is growing significantly.^3^, ^4^, ^5^ Elderly patients represent a high‐risk group, with studies reporting significantly higher morbidity and mortality compared to younger cohorts.^6^, ^7^, ^8^ Beyond the specific risks for hepatectomy in elderly patients, previous studies of all abdominal surgeries have also suggested that operative morbidity and mortality dramatically increases following age 75.^9^ With growing application of hepatic resection in elderly patients, optimizing outcomes in these challenging cases represents an important consideration for hepatobiliary surgeons.

Recent studies, most notably two recent systematic reviews and one large multicenter study, have suggested that minimally invasive (MIS) approaches to hepatectomy may provide superior outcomes compared to open surgery for geriatric patients.^10^, ^11^, ^12^ These studies have cited reduced rates of serious complications, reduced length of stay, and equivalent oncologic outcomes. However, comparing open and laparoscopic hepatectomy, even with methods of matching, remains difficult due to heterogeneity of surgical considerations in such cases. Previous studies remain limited by small sample sizes; additionally, few studies have matched patients on surgical approach or biliary reconstruction.^10^, ^11^, ^12^ Larger, multicenter study of these high‐risk patients that controls for surgical considerations are needed to guide application of MIS hepatectomy in this cohort of patients.

The aim of this study was to evaluate demographic and outcome differences between geriatric patients receiving MIS compared to open hepatectomy using the National Surgical Quality Improvement Program (NSQIP) database. Outcomes will assist in guiding surgical decision making in these patients who are high‐risk for hepatic resection.

## 2 METHODS

### 2.1 Data source

To accomplish this study, the NSQIP hepatic‐targeted participant use data and general database for years 2016–2021 were merged. The NSQIP database collects data from the preoperative period and up to 30‐day postoperatively for patients undergoing surgery. Data is collected across 874 hospitals in Canada and the United States. This study was exempt from ethics approval.

### 2.2 Study design, patient population, and variable definitions

This study represents a retrospective analysis of prospectively collected NSQIP data. We identified patients undergoing any hepatectomy using current procedural terminology (CPT) codes 47120 (hepatectomy, partial lobectomy), 47125 (left lobectomy), 47130 (right lobectomy), and 47122 (trisegmentectomy). For this study, only patients aged ≥75 were included. Age ≥75 was chosen for this study as this was previously shown to represent a specific age cut‐off for increased risk following major abdominal surgery.^9^

The primary objective of this study was to comparatively evaluate MIS compared to open hepatectomy in patients ≥75 years old in terms of demographics and early 30‐day outcomes.

We first characterized patients according to the type of surgery that they underwent (partial lobectomy, right lobectomy, left lobectomy, or trisegmentectomy). Additionally, we characterized surgical approach based on whether patients received biliary reconstruction (i.e., hepaticojejunostomy) or not. In terms of demographics, patients were further age, sex, body mass index (BMI), functional status before surgery (independent, partially dependent, unknown) and their American Society of Anesthesiologists (ASA) score. Patient comorbidities were compared including cardiopulmonary conditions such as congestive heart failure (CHF), hypertension, chronic obstructive pulmonary disease (COPD), and active smoking status. Other demographics included presence of diabetes, dialysis dependence, chronic steroid use, and diagnosis of preoperative bleeding disorder. Oncologic factors characterized in this study were preoperative neoadjuvant therapy, tumor size, and tumor invasion (described as T3 or T4 tumor status). Finally, preoperative sepsis (none, systemic inflammatory response syndrome, sepsis) was described.

Outcomes were defined according to NSQIP predetermined definitions.^13^ In this study we evaluated operative time, length of postoperative hospital stay, discharge destination (home, rehabilitation, increased level of care), unplanned reoperations, readmission to hospital within 30 days, and if the patients were still admitted to hospital >30 days postoperatively. Infectious postoperative complications were extracted including wound complications (superficial surgical site infection (SSI), deep SSI, organ space SSI, wound disruption/dehiscence), pneumonia and urinary tract infection. Unique to the NSQIP hepatic data, we also evaluated any postoperative bile leak, including any bile leak requiring drain maintenance, percutaneous drain placement, or reoperation. We also characterized any posthepatectomy liver failure and graded these complications according to the International Study Group of Liver Surgery.^14^ Postoperative bleeding was defined as any bleeding (requiring ≥1 blood transfusion). Serious complications included respiratory complications (unplanned intubation, pulmonary embolism), cardiovascular complications (myocardial infarction, cardiac arrest), acute renal failure, sepsis, septic shock, deep vein thrombosis (DVT), and cerebral vascular accidents. We also considered overall serious complications (presence of ≥1 serious complication as defined above, reoperation, still in hospital >30 days, bile leak requiring percutaneous or operative intervention, or Grades B and C posthepatectomy liver failure) and mortality within 30 days of operation.

In addition to NSQIP defined outcomes our study also defined outcomes according to the Clavien Dindo Classification (CDC)^15^ and using the updated Comprehensive Complications Index (CCI).^16^ The CCI was used because recent studies have supported the CCI as a superior measure of complications and associated with long‐term oncologic outcomes following hepatectomy.^17^, ^18^, ^19^ To calculate these scores complications were assessed and treatments were assumed based on accepted treatments for these complications as described in Table S1.

### 2.3 Statistical analysis

Due to large sample sizes data was assumed to be normally distributed and was presented as mean ± standard deviation for continuous variables and differences between groups evaluated using analysis of variance. Categorical variables were presented as counts and percentages, with differences analyzed using chi squared tests. Significance was considered as *p* < 0.05.

In the included patients (age ≥75) multivariable logistic modeling was completed to evaluate factors associated with serious complications and mortality. Within these models, MIS (compared to open) was include as a factor to evaluate the impact of surgical approach. To establish models a hypothesis driven purposeful methodology was applied with univariate analysis first conducted including both clinically relevant variables, and factors with a *p* < 0.10 included to generate a preliminary main effects logistic regression model. Collinearity was evaluated using the variance inflation factor (VIF), with >10 prompting exclusion from the model. The Brier score (BS) and receiver operating characteristic (ROC) curves were used to assess goodness of fit of the models with analysis conducted using Stata 17 (STATACorp LP).

In addition to the multivariable models a propensity score matched (PSM) assessment was completed. To accomplish these patients open hepatectomy were matched via PSM using probit treatment model to those undergoing MIS hepatectomy controlling for factors determined in the multivariable modeling with *p* < 0.1. Replacements were allowed with patients in the MIS group being allowed to match more than once to open approach patients to improve the propensity matching. An average estimate of treatment effect in the treated analysis was completed with results provided as the mean difference between MIS compared to open with 95% confidence intervals (95CI).

## 3 RESULTS

### 3.1 Demographic variables

We included 2674 patients undergoing hepatectomy in this study with 681 (25.5%) receiving MIS surgery. Importantly, there was a significant difference in the procedure performed open and MIS, with MIS approaches having more partial lobectomy (85.9% vs. 61.7%; *p* < 0.001). Additionally, fewer patients in the MIS group required biliary reconstruction (1.6% vs. 10.6%; *p* < 0.001). Patients undergoing MIS resection were older (79.3 vs. 78.7 years; *p* < 0.001) and had a higher rate of COPD (7.9% vs. 5.6%; *p* = 0.029). Otherwise, cohorts were similar with regards to sex, BMI, functional status, ASA classification, and other comorbidities (Table 1). Patients undergoing MIS and open approach had a similar rate of >10% weight loss in the 6 months before surgery but more patients receiving open surgery underwent neoadjuvant therapy (25.0% vs. 12.5%; *p* < 0.001). MIS resections were less likely to be completed on tumors with invasion (i.e., T3 or T4; 14.1% vs. 24.8%; *p* < 0.001) but tumor sizes were similar between cohorts (Table 1).

**Table 1:** Demographics of patients ≥75 years old undergoing hepatectomy comparing those receiving minimally invasive and open approaches.

	Open	Minimally invasiven	*p*‐value
	*n* = 1991	*n* = 683	
	*n* (%)	*n* (%)	
Surgery performed			***<0.001***
Liver wedge resection	1226 (61.7)	587 (85.9)	
Left hepatectomy	231 (11.6)	47 (6.9)	
Right hepatectomy	348 (17.5)	33 (4.8)	
Trisegmentectomy	186 (9.3)	16 (2.3)	
Biliary reconstruction	211 (10.6%)	11 (1.6%)	***<0.001***
Age, years (mean ± *SD*)	78.7 (3.4)	79.3 (3.7)	***<0.001***
Female sex	831 (41.7)	312 (45.7)	0.072
BMI in Kg/m^2^ (mean ± *SD*) Kg/m^2^	27.2 (5.1)	27.5 (5.2)	0.160
Functional status			0.693
Independent	1944 (97.6)	665 (97.4)	
Partially dependent	37 (1.9)	16 (2.3)	
Totally dependent	2 (0.1)	(0.0)	
ASA category			0.074
1	1 (0.1)	1 (0.2)	
2	232 (11.7)	98 (14.4)	
3	1534 (77.2)	494 (72.4)	
4	219 (11.0)	89 (13.1)	
5	0 (0.0)	0 (0.0)	
Smoker	124 (6.2)	40 (5.9)	0.727
Diabetes			
No or diet controlled	1497 (75.2)	492 (72.0)	0.168
Non‐insulin dependent	347 (17.4)	141 (20.6)	
Insulin dependent	147 (7.4)	50 (7.3)	
Severe COPD	111 (5.6)	54 (7.9)	**0.029**
CHF	23 (1.2)	8 (1.2)	0.973
Hypertension	1397 (70.2)	523 (76.6)	0.001
Sepsis			0.846
None	1964 (98.6)	676 (99.0)	
SIRS	17 (0.9)	5 (0.7)	
Sepsis	7 (0.4)	1 (0.2)	
Dialysis dependent	5 (0.3)	5 (0.7)	0.076
Chronic steroid use	70 (3.5)	23 (3.4)	0.855
Bleeding disorder	84 (4.2)	36 (5.3)	0.252
Weight loss >10% months	100 (5.7)	22 (3.8)	0.082
Neoadjuvant	492 (25.0)	85 (12.5)	***<0.001***
Invasion	221 (24.8)	44 (14.1)	***<0.001***
Tumor size			0.814
<2 cm	193 (9.7)	73 (10.7)	
2–5 cm	412 (20.7)	134 (19.6)	
>5 cm	238 (12.0)	78 (11.4)	
Not reported	1148 (57.7)	398 (58.3)	

*Note*: Bold and italic *p*‐values represent statistical significance according to an alpha of 0.05.

Abbreviations: ASA, American Society of Anesthesiologists; BMI, body mass index; COPD, chronic obstructive pulmonary disease.

### 3.2 Postoperative outcomes bivariate analysis

Unadjusted evaluation of early (30 days) postoperative outcomes demonstrated that MIS approaches had a shorter operative duration (204.6 vs. 231.5 min; *p* < 0.001) and shorter LOS (4.5 vs. 7.6 days; *p* < 0.001; Table 2) compared to open surgery. Additionally, MIS hepatectomy had fewer complications on nearly all measured domains including the rate of prolonged hospital admission (i.e., >30 days), readmission, reoperation, infectious complications, and cardiac complications (Table 2). Patients undergoing MIS hepatectomy were more likely to be discharged home (90.6% vs. 80.0%; *p* < 0.001) rather than rehab or to skilled care, and also had a significantly lower rate of postoperative liver failure (3.1% vs. 7.8%) or serious complications (8.8% vs. 18.7%; *p* < 0.001). Evaluating complications, patients undergoing MIS hepatectomy had significantly lower CCI (7.2 vs. 14.2; *p* < 0.001) and fewer complications of all CDC grades, with the biggest difference being for CDC grade 2 complications (13.8% vs. 26.2%; *p* < 0.001; Table 2). There was no significant differences in postoperative strokes, MI, or mortality (Table 2).

**Table 2:** Thirty‐day postoperative outcomes for patients ≥75 years old undergoing hepatectomy comparing those receiving minimally invasive and open approaches.

	Openn	Minimally invasiven	*p*‐value
	*n* = 1991	*n* = 683	
	*n* (%)	*n* (%)	
Operative duration (minutes; mean ± *SD*)	231.5 (108.9)	204.6 (126.1)	***<0.001***
Length of stay (days; mean ± *SD*)	7.6 (5.4)	4.5 (3.9)	***<0.001***
Unplanned reoperation	83 (4.2)	11 (1.6)	***0.002***
Discharge destination			
Home	1593 (80.0)	619 (90.6)	
Rehab	92 (4.6)	16 (2.3)	***<0.001***
Skilled care, not home	144 (7.2)	25 (3.7)	
Other	162 (8.1)	23 (3.3)	
Still in hospital >30 days	57 (2.9)	4 (0.6)	***0.001***
Readmission	28 (11.5)	56 (8.2)	***0.017***
Superficial SSI	75 (3.8)	12 (1.8)	***0.011***
Deep SSI	5 (0.3)	1 (0.2)	0.618
Organ space SSI	206 (10.4)	28 (4.1)	***<0.001***
Wound disruption	16 (0.8)	1 (0.2)	0.062
Sepsis	99 (5.0)	11 (1.6)	***<0.001***
Septic shock	69 (3.5)	4 (0.6)	***<0.001***
Pneumonia	124 (6.2)	24 (3.5)	***0.007***
Unplanned intubation	95 (4.8)	17 (2.5)	***0.010***
DVT	41 (2.1)	4 (0.6)	***0.010***
Pulmonary embolism	21 (1.1)	7 (1.0)	0.947
Acute renal failure	45 (2.3)	6 (0.9)	***0.023***
Urinary tract infection	68 (3.4)	13 (1.9)	***0.047***
Cerebral vascular accidents	7 (0.4)	2 (0.3)	0.819
Myocardial infarction	49 (2.5)	15 (2.2)	0.696
Cardiac arrest	38 (1.9)	4 (0.6)	***0.016***
Liver failure grade			
A	48 (2.4)	10 (1.5)	
B	55 (2.8)	4 (0.6)	***<0.001***
C	53 (2.7)	7 (1.0)	
Liver failure (any grade)	156 (7.8)	21 (3.1)	***<0.001***
Bleed	465 (23.4)	73 (10.7)	***<0.001***
Serious complication	373 (18.7)	60 (8.8)	***<0.001***
Comprehensive complication index (mean ± *SD*)	14.3 (20.1)	7.2 (5.3)	***<0.001***
Clavien Dindo complications			< ***0.001***
1	87 (4.4)	27 (4.0)	
2	518 (26.2)	94 (13.8)	
3A	53 (2.7)	10 (1.5)	
3B	46 (2.3)	8 (1.2)	
4A	90 (4.5)	22 (3.2)	
4B	96 (4.9)	9 (1.3)	
Mortality	8 (1.4)	20 (1.7)	0.635

*Note*: Bold and italic *p*‐values represent statistical significance according to an alpha of 0.05.

Abbbreviations: MI, myocardial infarction; POPF, postoperative pancreatic fistula; SSI, surgical site infection; VTE, venous thromboembolism.

### 3.3 Predicting serious complications and mortality after controlling for demographic and surgical differences

Significant differences existed between patients undergoing open and MIS hepatectomy and multivariable logistic regression modeling was completed to control for these differences, including operation type. Generated multivariable models evaluating factors associated with serious complications (ROC: 0.746 BS: 0.133) and mortality (ROC: 0.770, BS: 0.046) were both reliably predictive.

Results demonstrated that a MIS approach compared to open for hepatectomy in patients ≥75 years old was not associated with a significantly lower likelihood of serious complications (OR: 0.77; *p* = 0.219; Table 3). These results were achieved after controlling for surgery type, whereby undergoing right hepatectomy (OR: 4.08; *p* < 0.001) and trisegmentectomy (OR: 2.83; *p* < 0.001) but not left hepatectomy (OR: 1.28; *p* = 0.328) were independently associated with a higher likelihood of serious complications compared to partial lobectomy (i.e., wedge resection). Notably, biliary reconstruction was also independently associated with serious complications (OR: 3.50; *p* < 0.001). Additionally, female gender was associated with a reduction in serious complications (OR: 0.66; *p* = 0.017), while non‐insulin dependent diabetes and preoperative sepsis increased the likelihood (Table 3).

**Table 3:** Multivariable logistic regression evaluating predictors of serious complications in patients ≥75 years old undergoing hepatectomy.

Risk factor	Odds ratio	95% Confidence interval	*p*‐value
Minimally invasive (compared to open)	0.77	0.50–1.17	0.219
Age	1.04	1.00–1.09	0.060
BMI	1.00	0.97–1.04	0.834
Female gender	0.66	0.47–0.93	***0.017***
COPD	1.07	0.57–1.99	0.836
CHF	1.76	0.52–5.97	0.364
Hypertension	1.06	0.72–1.56	0.757
Diabetes			
Non‐insulin dependent	1.91	1.14–3.23	***0.015***
Insulin dependent	1.34	0.93–2.04	0.109
Smoking	0.97	0.52–1.82	0.932
Dialysis	–	–	–
Steroid	0.60	0.23–1.52	0.282
Bleeding disorder	1.12	0.54–2.29	0.766
Preoperative sepsis	5.11	1.62–16.12	***0.005***
Preoperative partially dependent (compared to independent)	1.18	0.47–3.00	0.725
Invasion	1.25	0.87–1.80	0.225
Surgery (compared to liver wedge resection)			
Left	1.28	0.78–2.12	0.328
Right	4.08	2.76–6.03	***<0.001***
Trisegmentectomy	2.83	1.73–4.62	***<0.001***
Biliary reconstruction	3.50	2.34–5.23	***<0.001***

*Note*: Brier score = 0.133. ROC area = 0.746. Dialysis dependence not included as it predicted failure perfectly.

Bold and italic *p*‐values represent statistical significance according to an alpha of 0.05.

Abbreviations: BMI, body mass index; CHF, congestive heart failure; COPD, chronic obstructive pulmonary disease; ROC, receiver operating characteristic.

Evaluation of factors independently associated with mortality demonstrated that MIS approach was not associated (OR: 1.19; *p* = 0.623; Table 4) with any mortality difference. Similar to complications, right hepatectomy was associated with increased likelihood of mortality (OR: 4.16; *p* < 0.001), while left hepatectomy (OR: 1.17; *p* = 0.739) and trisegmentectomy (OR: 1.39; *p* = 0.512) were not. Again, receiving biliary reconstruction was independently associated with mortality (OR: 3.66; *p* < 0.001). Non‐insulin dependent diabetes, preoperative bleeding disorder, and preoperative sepsis were also associated with mortality (Table 4).

**Table 4:** Multivariable logistic regression evaluating predictors of mortality in patients ≥ 75 years old undergoing hepatectomy.

Risk factor	Odds ratio	95% Confidence interval	*p*‐value
Minimally invasive (compared to open)	1.19	0.59–2.43	0.623
Age	1.05	0.97–1.13	0.255
BMI	0.99	0.94–1.05	0.830
Female gender	0.62	0.34–1.14	0.121
COPD	0.54	0.15–1.91	0.342
CHF	3.91	0.88–17.43	0.074
Hypertension	1.05	0.52–2.12	0.893
Diabetes			
Non‐insulin dependent	3.23	1.48–7.06	***0.003***
Insulin dependent	1.70	0.88–3.28	0.117
Smoking	1.39	0.51–3.82	0.524
Dialysis	–	–	–
Steroid	0.40	0.05–3.12	0.379
Bleeding disorder	2.78	1.04–7.42	***0.042***
Preoperative sepsis	7.91	2.10–29.81	***0.002***
Preoperative partially dependent (compared to independent)	0.24	0.03–2.15	0.200
Invasion	0.75	0.38–1.49	0.412
Surgery (compared to liver wedge resection)			
Left	1.17	0.46–2.99	0.739
Right	4.16	2.16–8.01	***<0.001***
Trisegmentectomy	1.39	0.52–3.71	0.512
Biliary reconstruction	3.66	1.89–7.10	***<0.001***

*Note*: Brier score = 0.046, ROC area = 0.770. Dialysis dependence not included as it predicted failure perfectly.

Bold and italic *p*‐values represent statistical significance according to an alpha of 0.05.

Abbreviations: BMI, body mass index; CHF, congestive heart failure; COPD, chronic obstructive pulmonary disease; ROC, receiver operating characteristic.

Factors included in PSM were determined based on MVR and controlled for diabetes preoperative sepsis, bleeding disorder, tumor invasion, and surgery type. Following PSM, assessment of outcomes demonstrated a nonsignificant reduction in serious complications (−1.9%; 95CI: −6.3% to 2.5%; *p* = 0.403) and no difference in mortality (0.9%; 95CI: −1.5% to 3.4%; *p* = 0.446). However, after PSM a significant reduction in LOS (−1.99 days; 95CI: −2.54 to −1.44 days; *p* < 0.001), CDC (−0.26 points; 95CI: −0.47 to −0.05; *p* = 0.016) and CCI (−2.79 points; 95CI: −5.17 to −0.41; *p* = 0.022) were noted. No difference existed for operative duration (9.88 min; 95CI: −5.73 to 25.49 min; *p* = 0.215).

Due to previous studies demonstrating significant reductions in serious complications with MIS approaches for geriatric patients without controlling for biliary reconstruction two post hoc analyzes were completed. First, we completed a multivariable logistic regression evaluating factors associated with serious complications but excluded biliary reconstruction in this model. This demonstrated that when not controlling for biliary reconstruction, MIS hepatectomy was significantly associated with reduced likelihood of serious complications (OR: 0.65; *p* = 0.043, presented in Table S2). The second post hoc analysis excluded all patients in this study with biliary reconstruction and performed multivariable modeling and PSM on this cohort of patients. This demonstrated nearly identical results as our controlled analyzes where MIS approaches were not associated with any difference in serious complications (OR: 0.73; 95CI: 0.47–1.15; *p* = 0.176) or mortality (OR: 1.00; 95CI: 0.46–2.21; *p* = 0.991) on multivariable analysis (Table S3). Also, similar results were achieved in this cohort following PSM with MIS approaches not having an impact on serious complications (−2.1%; 95CI: −6.5% to 2.4%; *p* = 0.370), or mortality (0.5%; 95CI: −2.2% to 3.4%; *p* = 0.683) but was associated with reduced LOS (−1.95; 95CI: −2.5 to −1.4; *p* < 0.001), CDC (−0.27; 95CI: −0.49 to 0.06; *p* = 0.014), and CCI (−2.86; 95CI: −5.38 to −0.34; *p* = 0.026).

## 4 DISCUSSION

This is the largest study to date comparing MIS and open hepatectomy in geriatric patients and overall suggests superiority of MIS approaches but tempers results from previous studies. Patients undergoing MIS hepatectomy have superior outcomes across nearly all domains and after demographic and surgical adjustment PSM analysis suggests a reduction in LOS, CDC, and CCI. However, as opposed to previous studies, after controlling for surgical and operative differences including biliary reconstruction no significant differences exist in terms of serious complications or mortality.

Our results suggest equal rates of serious complications between MIS and open approaches and contradict findings reported by two recent systematic reviews and a more recent large multicentre study who suggested that MIS approaches in elderly patients resulted in fewer serious complications.^12^, ^20^, ^21^ However, these results require further context to explain these differences. Notably, our study demonstrates significant differences in biliary reconstruction rates between groups and controls for this in both multivariable and PSM analysis. By comparison, the recent multicentre study by Sijberden et al.^12^ did not match based on biliary reconstruction and only 2 of 12 studies included in the most recent systematic review reported matching based on biliary reconstruction.^10^, ^11^ In our multivariable modeling biliary reconstruction represents the single largest independent predictor of serious complications and mortality and should therefore be controlled for when comparing patients. None of the studies that included biliary reconstruction as a controlled factor found differences in serious complications. Additionally, post hoc analysis of our data excluding biliary reconstruction as a controlled factor in multivariable logistic analysis demonstrated significant reduction in serious complications with MIS compared to open approaches. Furthermore, following a completely separate analysis of our data excluding patients with biliary reconstruction also demonstrated no significant impact of MIS approaches on serious complications. Overall, when better controlling for surgical differences and biliary reconstruction there does not appear to be any significant difference in serious complications or mortality between MIS and open approaches to hepatectomy in geriatric patients.

Despite similar rates in serious complications the benefits of MIS hepatectomy should not be overlooked. Our results continue to support previous literature showing reduction in LOS and a reduction in CDC and CCI with MIS approaches.^10^, ^11^, ^12^, ^20^, ^21^ Considering previous studies and our own these findings remain consistent, highlighted by *I* ^2^ values of 0% in both prior systematic review. The reduction in CDC and CCI support a reduction in overall complications even after controlling for biliary reconstruction. For elderly patients, often with concurrent frailty, even small complications can lead to delirium and substantial morbidity as evidenced by prolonged LOS with open operations and should be highlighted as a possible benefit of MIS approaches. Additionally, considering recent literature supporting an association between CCI and long‐term oncologic survival it is potentially promising that MIS hepatectomy may provide equivalent long‐term outcomes.^17^, ^18^, ^19^ More convincingly, Sijberden et al.^12^ demonstrates similar 1, 3, and 5‐year overall and disease‐free survivals, which should reassure surgeons who have been early adopters of MIS hepatectomy. Taken together, and in the context of better controlling for operative considerations, results from this study suggest that MIS hepatectomy offers improved outcomes for geriatric patients but that results from prior studies should be tempered.

Briefly, an important additional finding worth discussing from this study is the rates of MIS hepatectomy in geriatric patients. In this study across >800 North American centers only 25.5% of patients ≥75 received MIS hepatectomy, yet only 11.8% of tumors were >5 cm and only 8.3% required biliary reconstruction. With these results and alongside findings from other centers a broader uptake of MIS hepatectomy for geriatric patients is warranted. Recent results suggest that the learning curve for these procedures may be as few as 30–50 for pioneers and 15–20 for second generation surgeons.^22^, ^23^, ^24^, ^25^ Studies have supported use of simulation‐based learning and gradual implementation of MIS techniques to reduce the learning curve and may help pioneers at specific centers adopt MIS hepatectomy.^25^ Meanwhile, Fellowship programs should aim to implement simulation and volume to enable trainees to acquire the skillset required to continue advancing our field. Overall, considering growing data to support MIS approaches in geriatric patients and growing geriatric patient population, having the skillset and availability of this approach across centers should be encouraged.

Limitations of this study are primarily related to use of a standardized database for data collection. Potentially most importantly, the NSQIP database does not collect granular data to better define the extent of surgical procedures including a lack of information on vascular reconstruction or concurrent procedures. It can be assumed that open procedures have a bias towards more complex resections and therefore, outcomes from this study may overestimate the benefits of MIS approaches. On the other hand, NSQIP collects data from all centers including variable MIS hepatectomy volumes and expertize with MIS approaches likely varies. In this case, for expert centers outcomes from MIS approaches may actually be superior than those reported here. In addition to these limitations this study represents a retrospective analysis and findings may be associated with unmeasured confounding factors.

Overall this study demonstrates that after adjusting for demographics and surgical technique (including biliary reconstruction) there is no difference in serious complications between MIS and open hepatectomy, tempering outcomes from prior studies. However, when considering all complications and LOS MIS hepatectomy continues to offer significant benefits for geriatric patients. While this approach may not be compatible with all resections or patients, training and availability across centers in MIS approaches may allow improved outcomes in this high risk cohort.

## 5 CONCLUSION

Compared to open hepatectomy, MIS surgical approaches reduce LOS and overall complications in geriatric patients undergoing hepatectomy. Additional long‐term oncologic evaluation of MIS approaches is needed but current safety data is encouraging and warrants broader uptake of MIS hepatectomy in geriatric patients.

## AUTHOR CONTRIBUTIONS

Kevin Verhoeff assisted with conceptualization, methodology, data curation and analysis, investigation, editing, and supervision. Douglas Quan, Juan Glinka, Ephraim S. Tang, and Anton Skaro assisted with conceptualization, methodology, manuscript editing, and supervision.

## CONFLICT OF INTEREST STATEMENT

The authors declare no conflicts of interest.

## SYNOPSIS

This study provides the largest evaluation of minimally invasive hepatic resection in elderly patients to date and provides important update on outcomes evaluating this topic in previous smaller studies. Results temper previously reported outcomes but support reduced LOS and complications with MIS approaches.

## Supporting information

Supporting information.

## Data Availability

All data from this study is available upon reasonable request to the corresponding author. American College of Surgeons National Surgical Quality Improvement Program and the hospitals participating in the ACS NSQIP are the source of the data used herein; they have not verified and are not responsible for the statistical validity of the data analysis or the conclusions derived by the authors.
