# Supplementary material for: Laparoscopic versus open hepatic resection in patients ≥75 years old: A NSQIP analysis evaluating 2674 patients
Source: J Surg Oncol. 2024 Aug 19;130(5):1006–13. doi: 10.1002/jso.27820 (PMC11654895; doi:10.1002/jso.27820)
Supplement: Supplementary file 1 — Supporting information. [file JSO-130-1006-s001.docx]

**Supplementary Material**

**Table S1.** Complications and their associated Clavien Dindo Classification applied during this study

| **Complication** | **Clavien Dindo Classification** |
| --- | --- |
| Superficial SSI | 1 |
| Deep incision SSI | 1 |
| Readmission | 1 |
| Bile leakage – spontaneous wound drainage | 2 |
| Bile leakage – drain maintained after post-operative day 3 | 2 |
| Liver failure Grade B | 2 |
| Organ space SSI | 2 |
| Pneumonia | 2 |
| Sepsis | 2 |
| DVT requiring therapy | 2 |
| Pulmonary embolism | 2 |
| Transfusion intraoperative or post-operative | 2 |
| Urinary tract infection | 2 |
| Bile leakage requiring percutaneous drain | 3A |
| Wound dehiscence | 3B |
| Return to OR | 3B |
| Cardiac arrest | 4A |
| Myocardial infarction | 4A |
| Acute renal failure | 4A |
| Unplanned intubation | 4A |
| Liver failure Grade C | 4B |
| Septic shock | 4B |
| Mortality | 5 |

**Table S2.** Multivariable logistic regression evaluating predictors of serious complications in patients ≥75 years old

| **Risk Factor** | **Odds Ratio** | **95% confidence interval** | **p-value** |
| --- | --- | --- | --- |
| Minimally invasive (compare to open) | 0.65 | 0.43 – 0.99 | ***0.043*** |
| Age | 1.03 | 0.98 – 1.08 | 0.212 |
| BMI | 1.00 | 0.97 – 1.03 | 0.844 |
| Female gender | 0.68 | 0.49 – 0.94 | ***0.021*** |
| COPD | 1.10 | 0.60 – 2.01 | 0.767 |
| CHF | 1.62 | 0.49 – 5.40 | 0.432 |
| Hypertension | 1.05 | 0.73 – 1.53 | 0.781 |
| Diabetes  Non-insulin Dependent  Insulin Dependent | 1.81  1.20 | 1.09 – 3.02  0.82 – 1.76 | ***0.023***  0.341 |
| Smoking | 0.91 | 0.49 – 1.69 | 0.772 |
| Dialysis | - | - | - |
| Steroid | 0.60 | 0.24 – 1.50 | 0.270 |
| Bleeding disorder | 1.08 | 0.54 – 2.18 | 0.828 |
| Preoperative Sepsis | 5.85 | 1.86 – 18.37 | ***0.002*** |
| Preoperative partially dependent (compared to independent) | 1.23 | 0.50 – 3.03 | 0.658 |
| Invasion | 1.24 | 0.87 – 1.77 | 0.234 |
| Surgery (compared to liver wedge resection)  Left  Right | 1.57  4.68 | 0.97 – 2.54  3.20 – 6.84 | ***0.068***  ***<0.001*** |
| Trisegmentectomy | 3.93 | 2.47 – 6.24 | ***<0.001*** |

Brier score = 0.139

ROC area = 0.719

BMI, body mass index; COPD, chronic obstructive pulmonary disease; CHF, congestive heart failure

**Table S3.** Multivariable logistic regression evaluating predictors of serious complications and mortality in patients ≥ 75 years old undergoing hepatectomy excluding patients who required biliary reconstruction during their surgery

| **Multivariable analysis evaluating factors associated with serious complications** | | | |
| --- | --- | --- | --- |
| **Risk Factor** | **Odds Ratio** | **95% confidence interval** | **p-value** |
| Minimally invasive (compared to open) | 0.73 | 0.47 – 1.15 | 0.176 |
| Age | 1.04 | 0.99 – 1.10 | 0.094 |
| BMI | 0.99 | 0.95 – 1.03 | 0.554 |
| Female gender | 0.63 | 0.42 – 0.92 | ***0.019*** |
| COPD | 0.74 | 0.35 – 1.58 | 0.440 |
| CHF | 1.67 | 0.46 – 6.02 | 0.432 |
| Hypertension | 1.20 | 0.76 – 1.89 | 0.268 |
| Diabetes  Non-insulin Dependent  Insulin Dependent | 1.98  1.27 | 1.11 – 3.54  0.83 – 1.96 | ***0.020***  0.109 |
| Smoking | 1.24 | 0.64 – 2.40 | 0.522 |
| Dialysis | - | - | - |
| Steroid | 0.43 | 0.12 – 1.49 | 0.184 |
| Bleeding disorder | 0.84 | 0.35 – 2.00 | 0.692 |
| Pre-operative Sepsis | 4.56 | 1.18 – 17.64 | ***0.028*** |
| Pre-operative partially dependent (compared to independent) | 1.04 | 0.35 – 3.08 | 0.944 |
| Invasion | 1.31 | 0.87 – 1.99 | 0.199 |
| Surgery (compared to liver wedge resection)  Left  Right | 1.30  4.00 | 0.73 – 2.33  2.59 – 6.18 | 0.378  ***<0.001*** |
| Trisegmentectomy | 2.89 | 1.58 – 5.30 | ***0.001*** |
| **Multivariable analysis evaluating factors associated with mortality** | | | |
| **Risk Factor** | **Odds Ratio** | **95% confidence interval** | **p-value** |
| Minimally invasive (compared to open) | 1.00 | 0.46 – 2.20 | 0.991 |
| Age | 1.03 | 0.94 – 1.13 | 0.552 |
| BMI | 0.99 | 0.92 – 1.06 | 0.825 |
| Female gender | 0.47 | 0.22 – 1.04 | 0.062 |
| COPD | 0.52 | 0.11 – 2.51 | 0.415 |
| CHF | 5.54 | 1.19 – 25.89 | ***0.029*** |
| Hypertension | 0.67 | 0.31 – 1.49 | 0.333 |
| Diabetes  Non-insulin Dependent  Insulin Dependent | 3.09  1.14 | 1.23 – 7.81  0.51 – 2.55 | ***0.017***  0.748 |
| Smoking | 1.64 | 0.56 – 4.78 | 0.363 |
| Dialysis | - | - | - |
| Steroid | 0.81 | 0.10 – 6.35 | 0.839 |
| Bleeding disorder | 2.19 | 0.67 – 7.16 | 0.194 |
| Pre-operative Sepsis | 12.76 | 2.50 – 62.51 | ***0.002*** |
| Pre-operative partially dependent (compared to independent) | 0.32 | 0.03 – 3.25 | 0.332 |
| Neoadjuvant | 0.62 | 0.17 – 2.19 | 0.454 |
| Invasion | 0.56 | 0.23 – 1.36 | 0.197 |
| Surgery (compared to liver wedge resection)  Left  Right | 0.95  4.53 | 0.26 – 3.40  2.17 – 9.45 | 0.935  ***<0.001*** |
| Trisegmentectomy | 1.01 | 0.21 – 4.81 | 0.990 |

BMI, body mass index; COPD, chronic obstructive pulmonary disease; CHF, congestive heart failure

Dialysis dependence not included as it predicted failure perfectly.
